# A descriptive study on feasibility of nasopharyngeal and oropharyngeal swab collection from pediatric research participants in Cebu, Philippines

**DOI:** 10.3389/fpubh.2025.1566688

**Published:** 2025-06-10

**Authors:** Clarissa De Guzman, Ma. Gladys Nicole Daque, March Helena Jane Lopez, Anna Maureen Cuachin, Maria Vinna Crisostomo, Michelle Ylade, Jacqueline Deen

**Affiliations:** Institute of Child Health and Human Development, National Institutes of Health, University of the Philippines-Manila, Manila, Philippines

**Keywords:** nasopharyngeal swab, oropharyngeal swab, pediatric, Cebu, Philippines

## Abstract

**Background:**

Nasopharyngeal (NPS) and oropharyngeal (OPS) swab collection are vital in the diagnosis and surveillance of respiratory viruses. However, the acceptability of these procedures among children remains a challenge.

**Methods:**

We conducted a descriptive study using data from two pediatric observational studies in Cebu, Philippines (July 2021–October 2022). One was a community-based study, involving febrile adolescents aged 13–19 years, and the other was a hospital-based study, involving febrile children aged 1 month to <5 years. Both studies aimed to collect NPS/OPS samples for respiratory pathogen testing, including SARS-CoV-2, influenza A/B, and respiratory syncytial virus. We described reasons for refusal of NPS/OPS collection obtained from parents or guardians who were approached for participation in these studies.

**Results:**

Among 180 children enrolled from study sites in Bogo and Balamban Cebu, 134 (74.4%) were from the community-based study and 46 (25.6%) from the hospital-based study. Twenty-nine (29/180; 16.1%) agreed to undergo NPS/OPS collection—all of whom were from the community-based study. None of the hospital-based participants agreed to undergo NPS/OPS collection as part of their participation in the study. Among the 151/180 (83.90%) participants who refused the research swab collection, 41 (27.2%) declined due to a prior swab, 31 (20.5%) cited fear or discomfort, and 28 (18.5%) felt it was unnecessary at the time.

**Conclusion:**

NPS/OPS collection was less acceptable in both community and hospital settings, unless mandated by local authorities based on the experience during the COVID-19 pandemic. Prior swabbing, procedural discomfort, and perceived lack of necessity were key barriers, especially among younger children and their caregivers.

## Introduction

The coronavirus disease 2019 (COVID-19) pandemic began in early 2020, prompting governments worldwide to implement lockdowns, quarantines, and other public health interventions to limit transmission. As the pandemic progressed, accurate and timely testing for severe acute respiratory syndrome coronavirus 2 (SARS-CoV-2), the causative agent of COVID-19, became critical for outbreak control. Testing enables the identification of new cases, contact tracing, and targeted isolation measures to reduce the spread of the virus (1).

Among the available diagnostic tools, real-time reverse transcriptase polymerase chain reaction (RT-PCR) using nasopharyngeal (NPS) and/or oropharyngeal (OPS) swabs remains the gold standard for detecting SARS-CoV-2 due to its high sensitivity and ability to detect infection within hours of specimen collection (2–4). These samples are obtained by inserting flocked swabs through the nostrils into the nasopharynx or through the mouth to reach the posterior oropharynx (3, 5). However, despite their diagnostic value, these procedures are often perceived as uncomfortable or distressing, particularly among children, contributing to test refusal (6, 7).

In pediatric populations, such refusals pose significant public health challenges. Children who forgo testing may miss timely diagnosis and treatment, contributing to missed cases and persistence of disease transmission in the household and even the community. Previous studies have explored testing barriers in adults, yet literature focusing on pediatric populations—especially in resource-limited and pandemic-intense settings—remains scarce.

This study aims to examine the acceptability of nasopharyngeal and/or oropharyngeal swab collection among children and their caregivers in hospital and community settings and to describe the most common reasons for testing refusal. Identifying procedure-related concerns and socio-behavioral factors influencing test acceptance is essential for improving diagnostic strategies, promoting child-friendly testing alternatives, and enhancing community participation in public health screening efforts (8).

## Materials and methods

This is a descriptive study using previously collected data from two pediatric observational studies conducted in Bogo City and Balamban municipality in Cebu, Philippines, from July 2021 to October 2022. The studies received ethical approval from the University of the Philippines—Manila (UPM) Research Ethics Board. Written informed consent was obtained from the parents or guardians of the participants, along with verbal assent from children when applicable.

One study was conducted in a community-based setting and involved children aged 13 to 19 years presenting to the study site with fever and respiratory symptoms. Upon obtaining informed consent, they were interviewed and NPS/OPS collection was conducted for confirmation of SARS-CoV-2 infection. Those who refused NPS/OPS swab collection were asked for their reason for refusal and these were recorded in the study chart.

The hospital-based study enrolled children more than 30 days to less than 5 years who presented to the hospital with similar symptoms. As mandated by the local COVID-19 guidelines at that time, nasopharyngeal and oropharyngeal swab would be obtained at the time of admission for SARS-CoV-2 RT-PCR test to triage incoming patients for admission to the COVID-19 ward. The results and specimen of these swab samples were not considered as part of the observational study’s research protocol since informed consent for participation in the study was not yet obtained during this time. Upon obtaining informed consent, parents or guardians of participants were interviewed and a second NPS/OPS collection procedure was requested where samples would be tested for respiratory pathogens including SARS-CoV-2, influenza A, influenza B, and respiratory syncytial virus as part of the objectives of the main study. During informed consent process, reasons for refusal were asked from the parents or guardians and these were recorded in the study charts. Participants and/or their caregivers who refused the research NPS/OPS procedure were not excluded from the main observational studies.

We extracted archived data on demographic, clinical characteristics and responses when offered a NPS/OPS collection for the two observational studies. Responses on reasons for refusal for the procedure were collated, reviewed and categorized into 10 major thematic categories during data analysis for this study. Categorical variables were compared using chi-squared or Fisher’s exact tests, and continuous variables were compared using t-tests. A *p*-value of <0.05 was considered statistically significant. Statistical analyses were performed using Stata version 17 (StataCorp LLC).

## Results

A total of 180 pediatric participants were included in this study: 134 (74.4%) from the community-based setting and 46 (25.6%) from the hospital-based setting. Of the total, only 29 participants (16.1%) agreed to nasopharyngeal and/or oropharyngeal swab collection, while 151 (83.9%) refused the procedure.

Nine (5.0%) of the children were less than 1 year of age. Thirty-seven (20.6%) were between 1 to 5 years old, and 134 (74.4%) were between the ages of 13 to 19 years. There were more females (93, 51.7%) than males (87, 48.3%).

We compared the characteristics between those who agreed and did not agree to provide an NPS/OPS sample (Table 1). All participants who agreed to the NPS/OPS were from the community-based study. Among these, 29 out of 134 (21.6%) parents or guardians consented to specimen collection. In contrast, none of the 46 participants (0%) in the hospital-based study agreed to the research swab collection. All participants, regardless of swab consent, agreed to provide clinical data and a blood sample.

**Table 1:** Comparison of characteristics.

Characteristic	Total (*n* = 180)	Community-based study (*n* = 134)	Hospital-based study (*n* = 46)	*p*-value
Age group				<0.0001
<1 year	9 (5.0%)	0 (0%)	9 (19.6%)	
1–5 years	37 (20.6%)	0 (0%)	37 (80.4)	
13–19 years	134 (74.4%)	134 (100%)	0 (0%)	
*Mean*	11.63 years	14.94 years	2.03 years	
*Range*	1 month to 19 years	13 to 19 years	1 month to 4 years	
*Minimum Age*	1 month old	13 years	1 month old	
*Maximum Age*	19 years	19 years	4 years	
Sex				<0.001
Female	93 (51.7%)	79 (59.0%)	14 (30.4%)	
Male	87 (48.3%)	55 (41.0)	32 (69.6%)	
Swab status				<0.001
Agreed to NPS/OPS	29 (16.1%)	29 (21.6%)	0 (0%)	
Refused NPS/OPS	151 (83.9%)	105 (78.4%)	46 (100%)	

**Figure 1:**
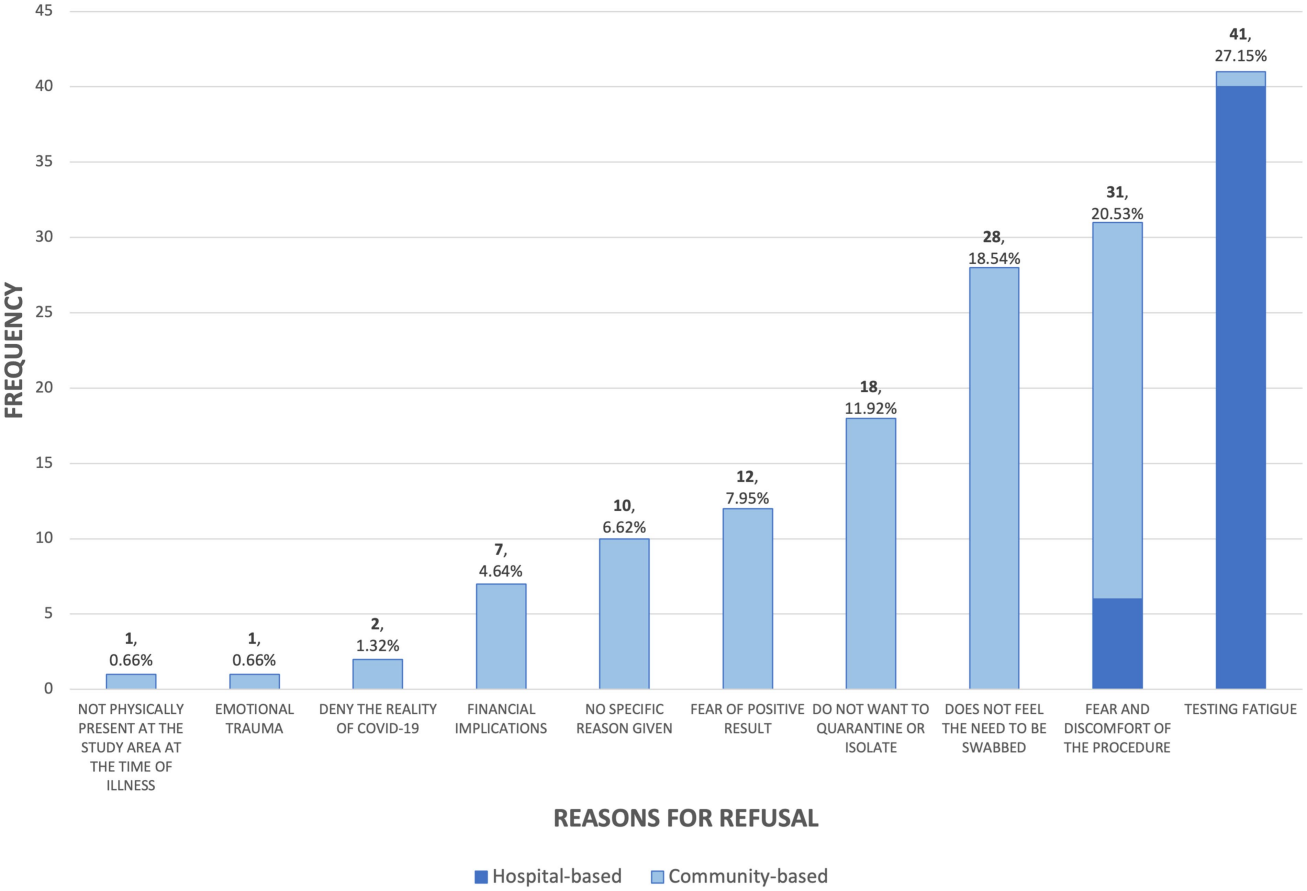
Reasons for refusal among respondents.

Reasons for refusal were documented and categorized into 10 major themes (Figure 1). The most frequently cited reason was testing fatigue or unwillingness to repeat the swab due to a prior specimen already collected (*n* = 41, 27.1%). Other commonly reported reasons included anxiety or fear of pain and discomfort of the procedure (*n* = 31, 20.5%), a perceived lack of necessity at the time of interview (*n* = 28, 18.5%), and avoidance of quarantine or isolation (*n* = 18, 11.9%). Additional reasons included concern on receiving a positive COVID-19 result (*n* = 12, 8.0%), fear of financial implications if they were to undergo quarantine—losing paid workings days and cost of medication (*n* = 7, 4.6%), denial of the reality of pandemic (*n* = 2, 1.3%), pandemic-related emotional trauma (*n* = 1, 0.7%), and unavailability of the child for specimen collection during the visit (*n* = 1, 0.7%). Ten respondents (6.6%) provided no specific reason but declined to undergo the procedure.

## Discussion

Our study revealed substantial hesitancy toward nasopharyngeal and/or oropharyngeal swab collection among children and their caregivers, particularly among hospitalized patients and very young children. Those who declined testing may be at increased risk for misdiagnosis, as COVID-19 symptoms overlap with other illnesses (9). The most frequently cited reasons for refusal included testing fatigue, procedural discomfort, perceived lack of necessity, fear of quarantine or isolation, and fear of receiving a positive result. Other reasons included financial burden, denial or disbelief in the pandemic’s reality, and emotional trauma associated with COVID-19. Similar reasons were reported in previous studies done in China (10) and Saudi Arabia (11), where financial difficulty and concerns over side effects were cited as causes for refusal.

There was complete refusal to undergo the procedure among the hospital-based group and were attributed to prior swabbing at admission, after which many children became distressed (e.g., crying or resisting), prompting parents or guardians to decline a second swab to avoid additional discomfort. Younger children were more likely to prefer non-invasive specimen collection methods, such as saliva testing, whereas older children tended to be more accepting of nasal swabs (12–15). Younger age was also associated with greater anxiety on the procedure (16). Testing procedures can negatively impact children, sometimes leading to aversive behaviors—especially when repeated testing is required (17). However, when procedures are painless, children and caregivers were more likely to participate in regular testing (2, 15, 18, 19).

In contrast to our findings, Gagnon (18) reported that nasopharyngeal swabs were not uncomfortable enough to deter caregivers from consenting to future tests. We speculate that unfamiliarity with the nasopharyngeal and oropharyngeal swab procedure—compared to more common procedures like blood collection—may have influenced decisions to refuse testing (11, 20). Additionally, refusal of medical procedures in pediatric care is not uncommon and is often driven by socio-economic and emotional factors, underscoring the need for improved communication and consent processes between healthcare providers and families (11, 20). The study done in Saudi Arabia similarly found that nasal swab collection was the most commonly refused pediatric procedure (11).

To address nasopharyngeal and oropharyngeal swab refusal, various alternatives have been proposed. Less invasive methods such as saliva testing and anterior nasal swabbing can minimize discomfort while still achieving diagnostic accuracy (2, 5, 12, 13, 15, 19). Saliva testing is particularly promising due to its non-invasive nature, ease of collection, and suitability for self- or caregiver collection (12, 13, 21). These alternatives may expand testing capacity, reduce the risk of viral transmission, and alleviate healthcare workforce burdens by limiting the need for trained personnel during collection (12, 21, 22).

Evidence also suggests that parent-collected (PC) nasal swabs perform comparably to nurse-collected (NC) swabs and may even yield more biological material (21). This may be due to parents’ familiarity with their children’s tolerances and their desire to minimize discomfort (21). During the pandemic, self- or parent-collected samples have helped maintain diagnostic accuracy while reducing the strain on healthcare systems (12, 21, 22). Given the discomfort associated with traditional NPS procedures (14, 22), alternatives like saliva and anterior nasal swabs offer better compliance among pediatric populations due to their simplicity, reduced invasiveness, and favorable tolerability (12, 13), while still providing reliable results (21).

Understanding test accuracy, cost-effectiveness, acceptability, and feasibility is essential to developing effective surveillance and testing strategies (12, 13, 18). Equally important is public education on the benefits and necessity of diagnostic testing (15, 16, 18, 23). Strategic communication can reduce procedural fear and stigma, foster trust, and increase testing acceptance (20). Given that acceptability varies across settings, tailored approaches are necessary.

Respiratory tract infections (RTIs) are a major cause of morbidity and hospitalization among children globally (24, 25). Accurate and rapid identification of pathogens is essential for prompt treatment, infection control, minimize unnecessary use of antibiotics, shortened hospital stays, and reduced overall healthcare costs (12, 13, 26). Nasopharyngeal and oropharyngeal swabs are the standard specimens for detecting respiratory pathogens, as the nasopharynx and oropharynx are common microbial entry points into the respiratory tract. However, their collection can be complex and poses practical challenges, especially among pediatric populations (12, 27). Despite their diagnostic value, the discomfort and procedural complexity associated with swab collection remain significant barriers to acceptance (13, 14). Understanding which clinical and diagnostic procedures are most frequently refused, along with the underlying reasons, is crucial for reducing refusal rates (11). In many healthcare settings (19), swab collection is now part of infection control protocols. Provision of psychological support and counseling to children and caregivers experiencing anxiety or stress related to testing may help mitigate refusal (11, 23).

This study has several limitations. First, findings may not be generalizable to all hospitals and communities in the Philippines due to the limited geographic scope and reliance on secondary data. Second, the hospital-based component included only a small number of children under five, limiting applicability to this age group. Third, since the study focused solely on pediatric patients, perceptions of nasopharyngeal and oropharyngeal swab among adults were not assessed. Lastly, the focus on febrile children with respiratory symptoms limits generalizability to asymptomatic or healthy individuals.

In summary, this study demonstrated that nasopharyngeal and/or oropharyngeal swab collection faced low acceptance in both hospital and community settings, largely due to procedure-related factors. Addressing barriers to testing—particularly those related to knowledge and perception—is essential. Identifying the most appropriate sampling method for COVID-19 tests in pediatric populations should be a research priority. Policymakers must consider family and patient preferences while balancing these with the diagnostic accuracy of various sampling techniques. Educational initiatives should aim to correct misconceptions and reduce stigma surrounding COVID-19 and its associated testing methods.

Public health messaging through traditional media, social media, and direct community outreach can counter misinformation and reduce testing-related stigma. Educational campaigns can be tailored at local, national, and global levels to promote accurate understanding of respiratory infections and the importance of diagnostic testing. Given the powerful role of social media in shaping perceptions, healthcare professionals should actively participate in online education to combat misinformation. As diagnostic testing remains central to managing the current and future respiratory pandemics, healthcare providers should encourage participation while respecting individual autonomy and prioritizing community well-being.

## Data Availability

The data analyzed in this study is subject to the following licenses/restrictions: data may be made available according to the University of the Philippines-Manila data sharing policy, upon request to the corresponding author. Requests to access these datasets should be directed to cadeguzman3@up.edu.ph.

## References

[1] Winter AK Hegde ST. The important role of serology for COVID-19 control. Lancet Infect Dis. (2020) 20:758–9. doi: 10.1016/S1473-3099(20)30322-4, PMID: 32330441 PMC7173803

[2] Thomas HM Mullane MJ Ang S Barrow T Leahy A Whelan A. Acceptability of OP/Na swabbing for SARS-CoV-2: a prospective observational cohort surveillance study in Western Australian schools. BMJ Open. (2022) 12:e055217. doi: 10.1136/bmjopen-2021-055217, PMID: 35082134 PMC8808315

[3] Zamboni M Drosten C Koopmans M Alland D Gao G Vandemaele K. Laboratory testing of 2019 novel coronavirus (2019-nCoV) in suspected human cases: interim guidance, (2020). Available online at: https://www.who.int/publications/i/item/laboratory-testing-of-2019-novel-coronavirus-(-2019-ncov)-in-suspected-human-cases-interim-guidance-17-january-2020 (Accessed on 2025 Jan 8).

[4] Liu R Han H Liu F Lv Z Wu K Liu Y. Positive rate of RT-PCR detection of SARS-CoV-2 infection in 4880 cases from one hospital in Wuhan, China, from Jan to Feb 2020. Clin Chim Acta. (2020) 505:172–5. doi: 10.1016/j.cca.2020.03.009 32156607 PMC7094385

[5] Frazee BW Alter H Chen CG Fuentes EL Holzer AK. Accuracy and discomfort of different types of intranasal specimen collection methods for molecular influenza testing in emergency department patients. Ann Emerg Med. (2018) 71:509–17. doi: 10.1016/j.annemergmed.2017.09.010 29174837

[6] Moisset X Gautier N Godet T Parabère S Pereira B Meunier E. Nasopharyngeal swab-induced pain for SARS-CoV-2 screening: a randomised controlled trial of conventional and self-swabbing. Eur J Pain. (2021) 25:924–9. doi: 10.1002/ejp.1722, PMID: 33394524

[7] Morris NP. Refusing testing during a pandemic. Am J Public Health. (2020) 110:1354–5. doi: 10.2105/AJPH.2020.305810, PMID: 32783720 PMC7427261

[8] Stangl AL Earnshaw VA Logie CH van Brakel WC Simbayi L Barré I. The health stigma and discrimination framework: a global, crosscutting framework to inform research, intervention development, and policy on health-related stigmas. BMC Med. (2019) 17:31. doi: 10.1186/s12916-019-1271-3 30764826 PMC6376797

[9] Larsen JR Martin MR Martin JD Kuhn P Hicks JB. Modeling the onset of symptoms of COVID-19. Front Public Health. (2020) 13:8. doi: 10.3389/fpubh.2020.00473 PMC7438535 32903584

[10] Wang Y r Jin R m Xu J w Zhang Z q. A report about treatment refusal and abandonment in children with acute lymphoblastic leukemia in China, 1997-2007. Leuk Res. (2011) 35:1628–31. doi: 10.1016/j.leukres.2011.07.004, PMID: 21802727

[11] Alharbi ES Alwabel AS Algaith NK Alqarzaee RS Alharbi RH Alkuraydis SF. Procedure and treatment refusal in pediatric practice: a single-center experience at a children’s hospital in Saudi Arabia. Cureus. (2025). Available online at: https://www.cureus.com/articles/145590-procedure-and-treatment-refusal-in-pediatric-practice-a-single-center-experience-at-a-childrens-hospital-in-saudi-arabia (Accessed on 2025 May 7). 10.7759/cureus.80936 PMC12009505 40255700

[12] Moreira VM Mascarenhas P Machado V Botelho J Mendes JJ Taveira N. Diagnosis of SARS-Cov-2 infection by RT-PCR using specimens other than Naso- and oropharyngeal swabs: a systematic review and Meta-analysis. Diagnostics. (2021) 11:363. doi: 10.3390/diagnostics11020363, PMID: 33670020 PMC7926389

[13] de Koff EM Euser SM Badoux P Sluiter-Post J Eggink D Sanders EAM. Respiratory pathogen detection in children: saliva as a diagnostic specimen. Pediatr Infect Dis J. (2021) 40:e351:–e353. doi: 10.1097/INF.0000000000003191, PMID: 34260500

[14] Hammitt LL Murdoch DR Scott JAG Driscoll A Karron RA Levine OS. Specimen collection for the diagnosis of pediatric pneumonia. Clin Infect Dis. (2012) 54:S132–9. doi: 10.1093/cid/cir1068 22403227 PMC3693496

[15] Schuster JE Potts J Selvarangan R Mast DK Goldman JL for the School TLC Study Group. A COVID-19 testing preference study in schools. Pediatrics. (2023) 152:e2022060352H. doi: 10.1542/peds.2022-060352h PMC10312271 37394509

[16] Aiano F Jones SEI Amin-Chowdhury Z Flood J Okike I Brent A. Feasibility and acceptability of SARS-CoV-2 testing and surveillance in primary school children in England: prospective, cross-sectional study. PLoS One. (2021) 16:e0255517. doi: 10.1371/journal.pone.0255517, PMID: 34449784 PMC8396768

[17] Slifer KJ Tucker CL Dahlquist LM. Helping children and caregivers cope with repeated invasive procedures: how are we doing? J Clin Psychol Med Settings. (2002) 9:131–52. doi: 10.1023/A:1014944110697

[18] Gagnon F Bhatt M Zemek R Webster RJ Johnson-Obaseki S Harman S. Nasopharyngeal swabs vs. saliva sampling for SARS-CoV-2 detection: a cross-sectional survey of acceptability for caregivers and children after experiencing both methods. PLoS One. (2022) 17, 7–8. doi: 10.1371/journal.pone.0270929, PMID: 35802720 PMC9269879

[19] Harwood. Comparison of the pain experienced with anterior nasal swabs and nose and throat swabs in children. Arch Dis Child. 107, 207. 34728461 10.1136/archdischild-2021-321708 PMC8785051

[20] Levy DL M Larcher V Kurz R Ethics Working Group of the Confederation of European Specialists in Paediatrics (CESP). Informed consent/assent in children. Statement of the ethics working Group of the Confederation of European specialists in Paediatrics (CESP). Eur J Pediatr. (2003) 162:629–33. doi: 10.1007/s00431-003-1193-z 12884032

[21] Woodall CA Thornton HV Anderson EC Ingle SM Muir P Vipond B. Prospective study of the performance of parent-collected nasal and saliva swab samples, compared with nurse-collected swab samples, for the molecular detection of respiratory microorganisms. Microbiol Spectr. (2021) 9:e00164–21. doi: 10.1128/Spectrum.00164-21 34756077 PMC8579848

[22] Spencer S Thompson MG Flannery B Fry A. Comparison of respiratory specimen collection methods for detection of influenza virus infection by reverse transcription-PCR: a literature review. J Clin Microbiol. (2019) 57, 4–5. doi: 10.1128/JCM.00027-19, PMID: 31217267 PMC6711916

[23] Walsh P Nguyen TA Higashida K Michaelson S Pham K Nguyen P. Do infants and toddlers prefer nasal swabs or washes for specimen collection? Pediatr Infect Dis J. (2010) 29:1156–7. doi: 10.1097/INF.0b013e3181fb45ae, PMID: 21099660

[24] Williams BG Gouws E Boschi-Pinto C Bryce J Dye C. Estimates of world-wide distribution of child deaths from acute respiratory infections. Lancet Infect Dis.. (2002) 2, 25–32. doi: 10.1016/s1473-3099(01)00170-0 11892493

[25] Niederman MS Krilov LR. Acute lower respiratory infections in developing countries. Lancet Lond Engl. (2013) 381:1341–2. doi: 10.1016/S0140-6736(12)62178-3 23369798

[26] Lee BR Hassan F Jackson MA Selvarangan R. Impact of multiplex molecular assay turn-around-time on antibiotic utilization and clinical management of hospitalized children with acute respiratory tract infections. J Clin Virol Off Publ Pan Am Soc Clin Virol. (2019) 110:11–6. doi: 10.1016/j.jcv.2018.11.006, PMID: 30502640 PMC7106386

[27] Ma ZY Deng H Hua LD Lei W Zhang CB Dai QQ. Suspension microarray-based comparison of oropharyngeal swab and bronchoalveolar lavage fluid for pathogen identification in young children hospitalized with respiratory tract infection. BMC Infect Dis. (2020) 20:168. doi: 10.1186/s12879-020-4900-8, PMID: 32087697 PMC7036252

